# Real-world data for precision public health of noncommunicable diseases: a scoping review

**DOI:** 10.1186/s12889-022-14452-7

**Published:** 2022-11-24

**Authors:** Oliver J. Canfell, Zack Kodiyattu, Elizabeth Eakin, Andrew Burton-Jones, Ides Wong, Caroline Macaulay, Clair Sullivan

**Affiliations:** 1 Centre for Health Services Research, Faculty of Medicine, The University of Queensland, St Lucia, QLD Australia; 2 UQ Business School, Faculty of Business, Economics and Law, The University of Queensland, St Lucia, QLD Australia; 3 Digital Health Cooperative Research Centre, Australian Government, Sydney, NSW Australia; 4 Health and Wellbeing Queensland, Queensland Government, The State of Queensland, Milton, QLD Australia; 5 Queensland Digital Health Centre, Faculty of Medicine, The University of Queensland, Herston, QLD Australia; 6 School of Clinical Medicine, Faculty of Medicine, The University of Queensland, St Lucia, QLD Australia; 7 School of Public Health, Faculty of Medicine, The University of Queensland, St Lucia, QLD Australia; 8 Department of Health, Office of the Chief Clinical Information Officer, Clinical Excellence Queensland, Queensland Government, Brisbane, QLD Australia; 9 Department of Health, Metro North Hospital and Health Service, Queensland Government, Herston, QLD Australia

**Keywords:** Public health, Health promotion, Preventive medicine, Public health informatics, Medical informatics, Chronic disease, Noncommunicable diseases

## Abstract

**Background:**

Global public health action to address noncommunicable diseases (NCDs) requires new approaches. NCDs are primarily prevented and managed in the community where there is little investment in digital health systems and analytics; this has created a data chasm and relatively silent burden of disease. The nascent but rapidly emerging area of precision public health offers exciting new opportunities to transform our approach to NCD prevention. Precision public health uses routinely collected real-world data on determinants of health (social, environmental, behavioural, biomedical and commercial) to inform precision decision-making, interventions and policy based on social position, equity and disease risk, and continuously monitors outcomes – the right intervention for the right population at the right time. This scoping review aims to identify global exemplars of precision public health and the data sources and methods of their aggregation/application to NCD prevention.

**Methods:**

The Preferred Reporting Items for Systematic Reviews and Meta-Analyses extension for scoping reviews (PRISMA-ScR) was followed. Six databases were systematically searched for articles published until February 2021. Articles were included if they described digital aggregation of real-world data and ‘traditional’ data for applied community, population or public health management of NCDs. Real-world data was defined as routinely collected (1) Clinical, Medication and Family History (2) Claims/Billing (3) Mobile Health (4) Environmental (5) Social media (6) Molecular profiling (7) Patient-centred (e.g., personal health record). Results were analysed descriptively and mapped according to the three horizons framework for digital health transformation.

**Results:**

Six studies were included. Studies developed population health surveillance methods and tools using diverse real-world data (e.g., electronic health records and health insurance providers) and traditional data (e.g., Census and administrative databases) for precision surveillance of 28 NCDs. Population health analytics were applied consistently with descriptive, geospatial and temporal functions. Evidence of using surveillance tools to create precision public health models of care or improve policy and practice decisions was unclear.

**Conclusions:**

Applications of real-world data and designed data to address NCDs are emerging with greater precision. Digital transformation of the public health sector must be accelerated to create an efficient and sustainable predict-prevent healthcare system.

**Supplementary Information:**

The online version contains supplementary material available at 10.1186/s12889-022-14452-7.

## Background

Globally, noncommunicable (chronic) diseases (NCDs) are responsible for approximately 70% of deaths worldwide [1]. A global syndemic – a synergy of multiple parallel epidemics – of NCDs, social inequity and COVID-19 is creating unprecedented global public health burden [2]. People are living more years in poor health despite medical and healthcare advancements in most countries [3]. The 2019 Global Burden of Disease Study concluded traditional public health practice is failing to address global increases in critical modifiable risk factors and recommended regular and granular reporting on population health is required to promote quick and decisive action [3–5].

Current best-practice public health leverages ‘traditional’ data that is created using specialised methods specifically for secondary use [6], often as single cross-sectional snapshots, including point-prevalence surveys, databases and disease registries (see Table 1) [7]. This data has significant utility for surveillance, epidemiological and planning purposes; however, it typically has a limited sample size and is refreshed at prolonged intervals (often years). One national example is the National Health Survey in Australia – a traditional health data asset. The 2018 Australian Burden of Disease report was released in August 2021 [8]; a minimum three-year latency period between data collection and availability to health system and public health stakeholders presents an unacceptable risk to the accuracy and timeliness of decisions, interventions and policy. Traditional data becomes outdated beyond the collection period and thus the evidence-base for decision-making and policy risks becoming obsolete.

**Table 1:** Data comparison between traditional public health and precision public health

**Domain**	**Traditional public health** *Current state*	**Precision public health** *Future state*
Definition	Use of data for surveillance and epidemiology to inform health policy, community interventions and target populations with disadvantage	Use of routinely collected data to inform precision policy, interventions and decision-making based on social position, equity and disease risk
Data sources	Designed by user specifically for secondary use	Real-world activities such as provision of acute care, wearable devices
Original intent of data	Secondary use	Routine activity
Data refresh	Years	Near or real-time
Data analytics	Descriptive	Descriptive, Predictive, Prescriptive

Precision public health is a modern iteration of traditional public health that uses traditional data together with routinely collected data to contemporaneously guide precision policy, interventions and decision-making based on social position, equity and disease risk to improve population health [9, 10]. This capability creates a responsive and agile ‘learning *public* health system’, where continuous, near real-time streams of data relevant for disease prevention and public health is used to improve decision accuracy and preventive care for future populations [7, 11]. One example in the USA, *RiskScape,* aggregates electronic health record (EHR) data in near real-time (~ 1 month) to review, analyse, map and trend data on chronic conditions and infectious diseases in 20% of the Massachusetts population [12]. These analytics are used by relevant state public health departments to monitor conditions of interest and plan interventions according to population risk. A similar proof-of-concept has emerged in Australia, *PopHQ,* a population health analytics tool that will leverage near real-time obesity data (~ 1 million patients) from a state EHR to help guide precision policy and practice at scale [13].

**Fig. 1:**
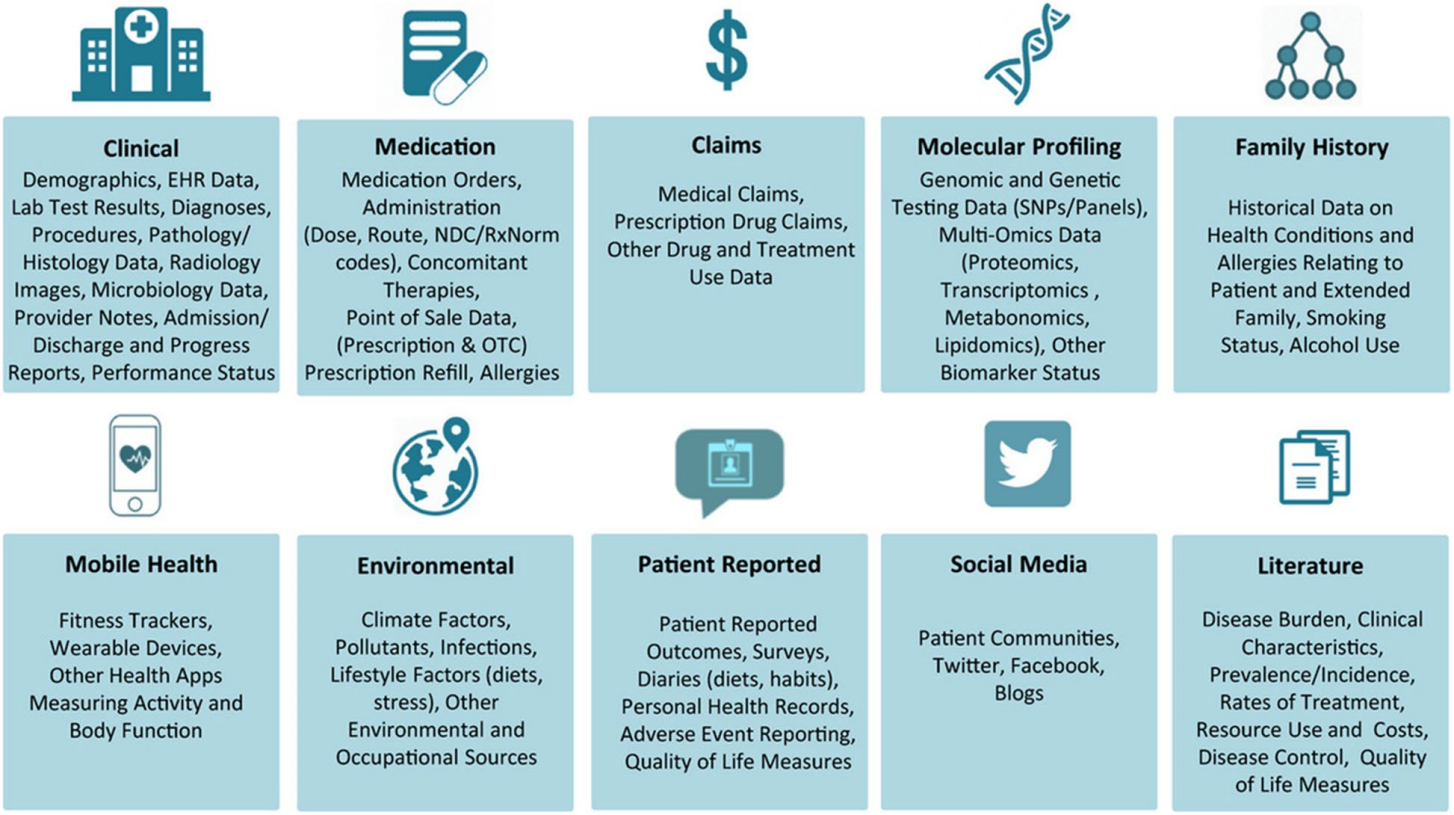
Types of real-world data (reproduced with permission from Swift et al*.* [15])

We are now living amidst a fourth, information-driven industrial revolution which has generated a new era of data, an asset that has been coined the new oil of the 21^st^ century [14]. Real world data is collected during routine activities (i.e., data not collected specifically for secondary use) and can unlock new information and evidence in near or real-time. (see Table 1). Real-world data is an umbrella term for routinely collected health data that is derived from outside traditional research and primary data collection settings (see Fig. 1) [15]. Some domains of real-world data (e.g., clinical, molecular profiling, mobile health and social media) are synonymous with big data as their sources generate massive, rich and continuously updating data.

Real-world data relevant to public health can be sourced from electronic medical/health records (EMRs/EHRs), personal health records (PHRs), insurance claims and billing databases, mobile health (mHealth) applications, wearables and sensors as part of the ‘Internet of Things’ (a connected network of physical objects), telecommunications data, social media and commercial transactions. Real-world data arising from the digital transformation of the acute healthcare sector (primarily via the EMR) is already reshaping healthcare delivery; rich and contemporaneous data is now available for every patient, every time and in real-time. This capability is being used to establish a learning health system where all data entered is reused to improve the care of subsequent patients [16]. EMRs are also beginning to digitally transform the public health sector. Recent systematic reviews have revealed that their enormous population reach, contemporaneity and clinical granularity is being leveraged for population health surveillance of communicable and noncommunicable diseases [17–20].

EMRs are one pillar of real-world data that can be leveraged as the digital foundation to advancing precision public health for NCDs. Timely and efficient decisions for NCD prevention require the complementary use of real-world data and traditional data. As an aggregated asset, these data create a single source of truth for all determinants of health relevant to NCDs: social, environmental, behavioural, biomedical and commercial. One jurisdiction (Queensland, Australia) has begun to systematically map all available data – real-world and traditional – that can be used to guide precision public health policies and interventions for obesity [21]. The social and behavioural determinants of health explain ~ 70% of health variance [22]; yet, surveillance has prioritised biomedical determinants as we cannot measure all non-biomedical determinants data contemporaneously and at population scale [7]. The primary research gap is our understanding of how real-world data and traditional data are being aggregated and used together to enable precision public health for NCDs.

This scoping review aims to answer the research question:

1. Globally, how is real-world data and traditional data being aggregated to support precision public health for NCDs?

Results of this review will be relevant to health system and public health decision-makers, policymakers, practitioners, managers, community leaders and researchers to mobilise real-world data and traditional data to advance precision public health for NCDs, create data-driven prevention services and build a sustainable healthcare system enabling healthier populations.

## Methods

### Design

We conducted our scoping review using systematic methodology (search strategy, selection criteria, data extraction and synthesis) according to Joanna Briggs Institute (JBI) guideline methodology for ‘systematic scoping reviews’ to ensure rigour and replicability [23]. Recent commentary has justified the growing role of scoping reviews when applied to the rapidly advancing and transdisciplinary nature of digital health and medicine [24]. We adhered to the Preferred Reporting Items for Systematic reviews and Meta-Analyses Extension for Scoping Reviews (PRISMA-ScR) checklist [25] (see Supplementary File 1). Our review protocol was made publicly available online with OSF Registries [26].

### Search strategy

A search strategy was created in consultation with a research librarian and three researchers (OC, ZK, CS), two of whom are subject matter experts (OC, CS). The search strategy was developed and implemented using the PCC (Population, Concept and Context) format [25]:

1. Population – Populations and Public Health
2. Concept – Digital aggregation
3. Context – Real-world and traditional data

The search strategy was developed in PubMed (see Table 2) and then translated to Scopus, EMBASE, IEEE Xplore, ACM Digital Library and Google Scholar for articles published in English from inception up to February 2021 (Supplementary File 2 presents the full search strategy for each database). We chose not to define NCDs in the (P) Population category due to the scoping nature of the review and as many NCDs are addressed singularly (e.g. cardiovascular disease (CVD), type 2 diabetes). Instead, articles were screened for application to NCDs according to the eligibility criteria.

**Table 2:** Search strategy in PubMed according to the Population, Concept, Context framework for scoping reviews [23, 25]

**Search category**	**Domain**	**PubMed**
Population	Population and public health	"population health"[MeSH Terms] OR "population health"[tiab] OR "public health"[MeSH Terms] OR "Public Health Surveillance"[MeSH Terms] OR "public health informatics"[MeSH Terms] OR "clinical frameworks"[tiab] OR "Learning Health System"[MeSH Terms] OR "Population Surveillance"[MeSH Terms] OR "surveillance"[tiab] OR "platform*"[ti] OR learning health system[tiab]
Concept	Digital aggregation	"data sharing"[tiab] OR "aggregat*”[Tiab] OR "Data network" OR linkage*[tiab] OR "data model*"[tiab]
Context	Real-world and traditional data	"Mobile Applications"[MeSH Terms] OR "social media"[MeSH Terms] OR "mobile health"[All Fields] OR "mobile technolog*"[All Fields] OR "mhealth"[tiab] OR "m-health"[tiab] OR "billing data"[tiab] OR "claims data"[tiab] OR "data aggregation*"[tiab] OR "health data"[tiab] OR survey*[tiab] OR "big data"[tiab] OR "digital health"[tiab] OR "Internet of Things"[MeSH Terms] OR "Internet of Things"[tiab] AND "medical records systems, computerized"[MeSH Terms] OR "Electronic Health Records"[MeSH Terms] OR "electronic health record*"[tiab] OR "EHR"[tiab] OR "electronic medical record*"[tiab] OR "EMR"[tiab] OR "medical records"[tiab]

### Study selection

After removal of duplicates via Endnote (phase 1) and Covidence (a web-based screening and data extraction tool for reviews) [27] (phase 2), title and abstract screening, full-text review and conflict resolution was conducted by two independent reviewers (OC, ZK) using the Covidence platform according to pre-defined eligibility criteria. A snowballing approach was implemented to identify potential additional articles – the reference lists of all articles that underwent full-text review were independently screened by two reviewers (OC, ZK).

### Eligibility criteria

Table 3 presents the inclusion and exclusion criteria for the present review. We consolidated some types of real-world data in the framework proposed by Swift et al [15]. to reflect their typical inclusion in a single data asset e.g. an EMR. We defined real-world data as routinely collected (1) Clinical, Medication and Family History (e.g. EMR) (2) Claims/Billing (3) Mobile Health (e.g. wearables/sensors/applications) (4) Environmental (e.g. lifestyle factors, retail data) (5) Social media (e.g. Twitter) (6) Molecular profiling (e.g. genomics) (7) Patient-centred i.e. EHR. Traditional data were defined as collected through specialised methods designed by the user, such as via surveys, registries, websites and administrative databases.

**Table 3:** Eligibility criteria for articles in the present review

**Inclusion criteria**	**Exclusion criteria**
• Article describes digital aggregation of real-world data and traditional data	• Only a single source of real-world data or traditional data is used
• The disease/s of interest is a noncommunicable (chronic) disease	• The disease/s of interest are communicable or acute
• Purpose of digital aggregation is applied, with the intent of contributing to improved community, population or public health	• Purpose of digital aggregation is for data linkage or research
	• Purpose of digital aggregation is applied to an individual patient or patient cohort for treatment or management of disease
	• Articles not published as full-text empirical studies (i.e. abstracts, conference proceedings, grey literature, dissertation or theses)

NCDs were defined according to aggregated definitions from the World Health Organisation (2013) [28] (excluding communicable diseases) to include:

- Major NCDs – cardiovascular diseases, cancers, chronic respiratory conditions and diabetes
- Other NCDs – renal, endocrine, neurological, haematological, gastroenterological, hepatic, musculoskeletal, metabolic (including obesity [29]) skin and oral diseases
- Mental health disorders
- Congenital (including genetic or chromosomal disorders) disorders
- Other conditions, disorders or disability originating from injury (e.g., limb amputation, traumatic brain injury)

### Data extraction and synthesis

**Fig. 2:**
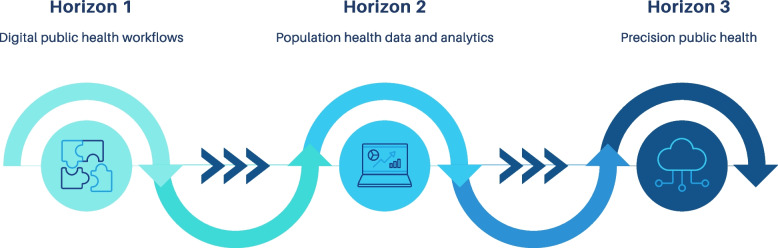
Three horizons for digital health transformation towards precision public health of noncommunicable disease [13]

A data extraction tool was customised in Covidence and specified according to the purpose of our scoping review. The following data were extracted from all included articles: (a) primary author and year of publication (b) country (c) aim (d) setting (e) population characteristics (f) target noncommunicable disease/s (g) types of real-world data (h) real-world data (i) traditional data (j) refresh frequency (k) aggregation of real-world data (l) analytics and (m) implementation. We performed two methods of data synthesis: (a) a narrative, descriptive synthesis and (b) mapping each included study to the three horizons roadmap for precision public health for NCDs [13] (see Fig. 2). Our discussion comments on the strengths and limitations of included studies and describes implications of these for advancing the field of precision public health for NCDs.

## Results

### Study selection

**Fig. 3:**
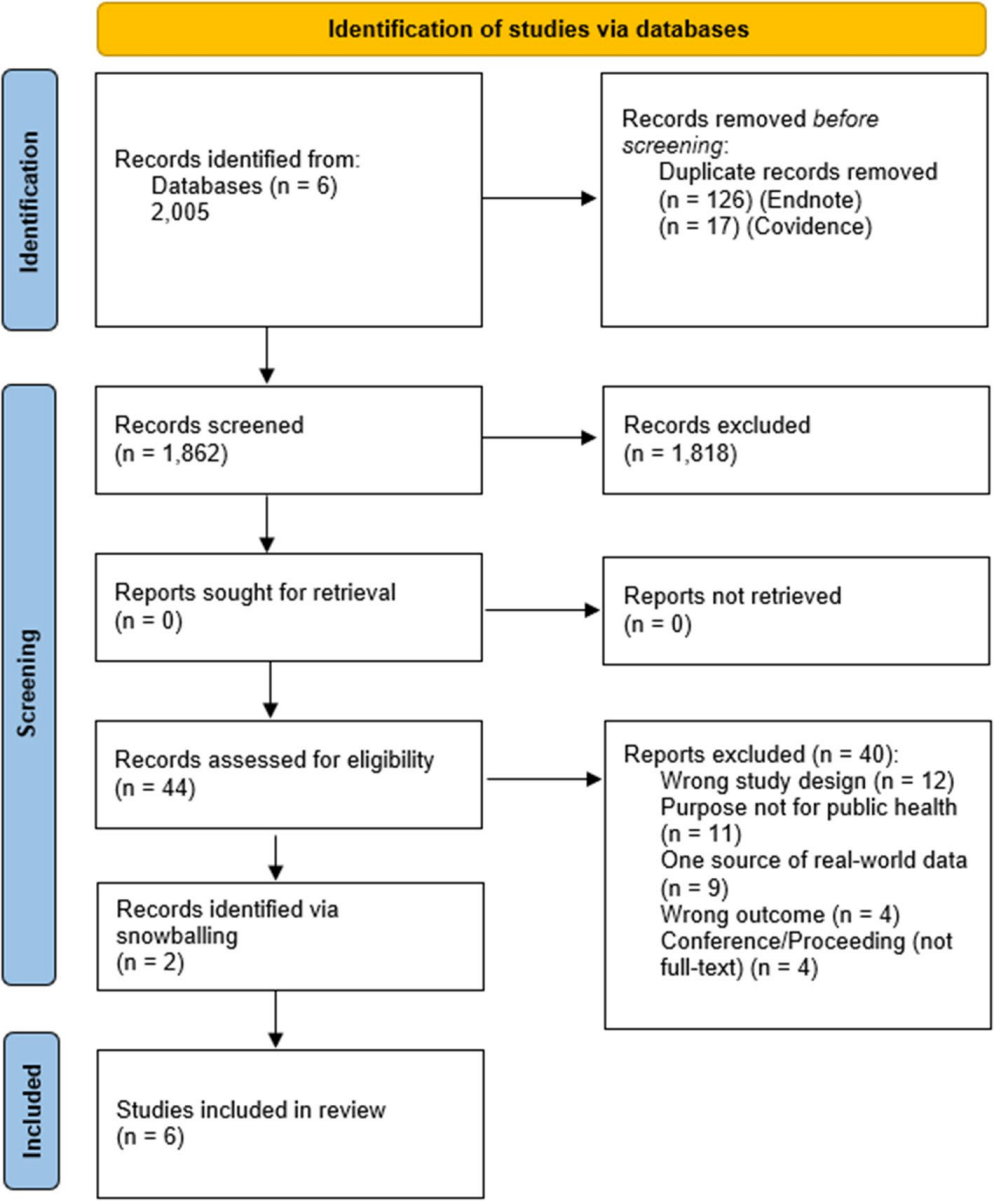
PRISMA flow diagram

The search yielded 2,005 articles from the databases PubMed, Scopus, EMBASE, IEEE Xplore, ACM Digital Library and Google Scholar (see Fig. 3 for the PRISMA flow diagram). After article duplicates were removed, 1,862 remained for title/abstract screening. A total of 44 articles were identified for full-text review. Six studies met our inclusion criteria and were included in the present review. Four of these studies were identified through the screening process and two were identified at full-text review via snowballing reference lists of screened articles.

### Study characteristics

Table 4 presents the characteristics of studies included in this review. Five studies were conducted in high-income countries; three were conducted in USA [30–32], two in Canada [33, 34] and one in China, uan upper-middle-income country [35]. Broadly, the objective of included studies was to describe the development of a population health surveillance platform. Studies aimed to (a) describe population surveillance methodology for people with CHD [30] (b) develop an EHR-based public health information exchange [31] (c) create a socially-generated health data model for disease surveillance (via *Social InfoButtons*) [32] (d) track the NCD epidemic in China *(NCDCMS)* [35] (e) describe development of a distributed model *(CCDSS)* for chronic disease surveillance in Canada [33] and (f) develop *PopHR*, a big data platform for population health surveillance in Canada [34].

**Table 4:** Characteristics of included studies

**Authors (Year)**	**Country**	**Aim**	**Setting**	**Population characteristics**	**Target chronic disease/s**
Glidewell et al. (2018) [30]	USA	To describe the surveillance methodology to better understand prevalence, care utilisation and outcomes of people with CHDs	*Academia/ Healthcare* 1. Emory University 2. Massachusetts Department of Public Health 3. New York State Department of Health	73,112 individuals (identified based on CHD codes in healthcare encounters)	Congenital Heart Disease
Guilbert et al. (2012) [31]	USA	To develop an EHR-based public health information exchange (via a new information system platform) to represent the ecologic health systems model	*Academia/ Healthcare / Government:* University of Wisconsin Dep't of Family Medicine Division of Public Health	40,320 children, 151,881 adults (total clinical sample)	Asthma, T2DM (use cases)
Ji et al. (2017) [32]	USA	To create a health data model and analytic framework that integrates and analyses openly available health data sources, particularly socially-generated data	*Academia:* New Jersey Institute of Technology City University of New York	17,407 (pts in SMN) 69,423 (posts in MedHelp) 86,715 reviews (WebMD)	MS, Fibromyalgia, MDD, GAD, CFS, ALS, Parkinson's, Epilepsy, SAD, Panic Disorder (top ten conditions)
Li et al. (2020) [35]	China	To develop a system (NCDCMS) for NCD surveillance and management	*Government:* Chinese Center for Disease Control and Prevention	Ningbo City (5.93 million) 201 medical facilities (98.1%)	T2DM, IHD, cerebrovascular disease, malignant and benign neoplasms of the central nervous system
Lix et al. (2018) [33]	Canada	To describe the process, structure, benefits and challenges of a distributed model for chronic disease surveillance (CCDSS)	*Government:* Public Health Agency of Canada (PHAC)	All Canadian provinces and territories	T2DM, HT, Mental illness, COPD, Asthma, IHD, AMI, HF, Osteoporosis, Parkinson's, MS, Stroke, Epilepsy, Dementia, Osteoarthritis
Shaban-Nejad et al. (2017) [34]	Canada	To develop PopHR, a big data platform for community and population health surveillance	*Academia/ Healthcare:* McGill University Tennessee Health Science Center	Census Metropolitan Area of Montreal, Quebec, Canada (25% of total population, ~ 1 million persons)	Arthritis, Asthma, Cancer, CKF, COPD, CHF, Mental illness, Obesity, IHD, MS, Parkinson's, T2DM

*CHD* Congenital heart disease, *EHR* Electronic health record, *T2DM* Type 2 diabetes mellitus, *MS* Multiple sclerosis, *MDD* Major depressive disorder, *GAD* Generalized anxiety disorder, *CFS* Chronic fatigue syndrome, *ALS* Amyotrophic lateral sclerosis, *SAD* Social anxiety disorder, *NCDCMS* Integrated noncommunicable disease collaborative management system, IHD Ischaemic heart disease, *CCDSS* Canadian Chronic Disease Surveillance System, *HT* Hypertension, *COPD* Chronic obstructive pulmonary disease, *AMI* Acute myocardial infarction, *HF* Heart failure, *CKF* Chronic kidney failure

Three of six studies were conducted in a partnered academia (university) and healthcare (health centres, department of health) setting [30, 31, 34]. Two studies were conducted in government settings [33, 35] (public health agencies) and one was conducted exclusively in academia (university) [32].

### Target population

The target population for included studies was large and varied. Three studies leveraged their sample from large geographical areas (Ningbo City [35], all Canadian provinces and territories [33], Montreal [34]) with populations ranging from 1 to 6 million persons. Two studies drew their samples from clinical health services (via EHRs) that ranged from 73,000 to 5 million persons [30, 35]. One study drew their sample from social media and government and health websites and quantified the sample in terms of patients, posts and reviews (~ 160,000 total) [32].

Five studies described digital aggregation of real-world data and traditional data to support targeting of 28 NCDs (e.g. asthma, type 2 diabetes mellitus, arthritis, cancer, chronic kidney failure, chronic obstructive pulmonary disease, mental illness, obesity). One study focused exclusively on congenital heart disease (CHD) [30].

### Characteristics of real-world and traditional data

Table 5 presents the characteristics, aggregation and application of real-world data and traditional data to support precision public health for NCDs. Four included studies leveraged ‘Clinical, Medication & Family Hx’ as the primary real-world data type [30, 31, 34, 35]. The data source generating this real-world data were organic and included an electronic health or medical record (EHR/EMR) [30, 31, 35] or public health insurance provider [34]. Data aggregation then occurred with real-world types of claims/billing [30, 34] and environmental (retail transactions) [34], and with traditional data via the country-specific Census [31, 34] (lifestyle factors, environmental characteristics), a disease registry (CHD) [30], electronic clinical history database [35] and administrative hospital discharge abstracts [33]. The social media platform ‘Twitter’ was aggregated with traditional health data via government and health websites and bibliographic databases (Centers for Disease Control, PubMed, WebMD, MedHelp) in *Social InfoButtons* [32].

**Table 5:** Characteristics, aggregation and application of real-world data and traditional data to support precision public health for noncommunicable diseases

**Authors (Year)**	**Country**	**Application**	**Target noncommunicable diseases/s**	**Types of RWD**	**RWD**	**Traditional data**	**Refresh frequency**	**Aggregation of RWD**	**Analytics**	**Implementation**
Glidewell et al. (2018) [30]	USA	-	Congenital Heart Disease	1. Clinical, Medication & Family Hx 2. Claims/Billing	1. EHR, birth and death files; SPARCS health information system 2. Medicaid, claims database	CHD registry	Static (cross-sectional)	Static records linked across data sources using Fine-Grained Records Integration and Linkage Tool and SAS. Microsoft Access database hosted standardised, aggregate data	Nil reported	Deidentified and deduplicated surveillance dataset transferred to CDC for review
Guilbert et al. (2012) [31]	USA	-	Asthma, T2DM (use cases)	Clinical, Medication & Family Hx	EHR	Census (social, economic and behavioural conditions)	Static (cross-sectional)	Built PHIN AVR Web Portal data system to aggregate EHR and community-level data	**Geospatial**—geocoding, spatial regression **Descriptive**—prevalence **Inferential**—multivariate analyses, data mining	Demographic, clinic, community disease summary reports
Ji et al. (2017) [32]	USA	Social InfoButtons	MS, Fibromyalgia, MDD, GAD, CFS, ALS, Parkinson's, Epilepsy, SAD, Panic Disorder (top ten conditions)	Social media	Twitter, SMN	CDC, PubMed, WebMD, MedHelp	Static (cross-sectional)	Data sources integrated via semantic web technology—links terms from different sources that describe the same concept	**Geospatial**—geocoding **Descriptive**—disease prevalence, social discussion, topic prevalence, associations, recommendations **Temporal**—treatment comparison over time	Platform 'Social InfoButtons'—government an end-user for disease surveillance and to increase awareness of social health trends
Li et al. (2020) [35]	China	NCDCMS	T2DM, IHD, cerebrovascular disease, malignant and benign neoplasms of the central nervous system	Clinical, Medications & Family Hx	EHR	Electronic clinical history database	Real-time (1 day)	Stepwise, bidirectional 3-level public health data exchange Uniform data standards required to connect HIS with NCDCMS	**Geospatial**—mapping	Implemented in 5 cities in Zhejiang Province
Lix et al. (2018) [33]	Canada	CCDSS	T2DM, HT, Mental illness, COPD, Asthma, IHD, AMI, HF, Osteoporosis, Parkinson's, MS, Stroke, Epilepsy, Dementia, Osteoarthritis	Claims/ billing	Health insurance registration, physician billing claims	Hospital discharge abstracts (via administrative dataset) and prescription drug records	Static (cross-sectional)	Provinces/territories generate aggregate data from PHAC data request. Data are reconciled based on uniform definitions	**Descriptive**—disease incidence, prevalence, mortality (bar and line charts) **Geospatial**—mapping across provinces and territories	Implemented by PHAC. CCDSS data produced in publications, disease reports and interactive open data resources
Shaban-Nejad et al. (2017) [34]	Canada	PopHR	Arthritis, Asthma, Cancer, CKF, COPD, CHF, Mental illness, Obesity, IHD, MS, Parkinson's, T2DM	1. Clinical, Medication & Family HX 2. Claims/Billing 3. Environmental	1. Public health insurance provider 2. Public health insurance provider 3. Retail transactions	Census and Surveys	Near real-time (2-weeks to 1-year)	Aggregated and individual-level data Server-client architecture: (1) Data processing (2) Data integration (3) Semantics	**Geospatial**—mapping **Descriptive**—bar charts, data tables, time series, scatter plots (w/ stratification) **Temporal**—time series, prevalence changes **Comparative**—intervention impact, multiple queries	Test users—public health and health service agencies Software verification, rapid feedback, usability testing Pilot implementation to support public health planning and health system management

*RWD* Real-world data, *CHD* Congenital heart disease, *EHR* Electronic health record, *SPARCS* Statewide Planning and Research Cooperative System, *CDC* Centers for Disease Control and Prevention, *T2DM* Type 2 diabetes mellitus, *PHIN* Private healthcare information network, *MS* Multiple sclerosis, *MDD* Major depressive disorder, *GAD* Generalized anxiety disorder, *CFS* Chronic fatigue syndrome, *ALS* Amyotrophic lateral sclerosis, *SAD* Social anxiety disorder, *NCDCMS* Integrated noncommunicable disease collaborative management system, *IHD* Ischaemic heart disease, *HIS* Health information system, *CCDSS* Canadian Chronic Disease Surveillance System, *HT* Hypertension, *COPD* Chronic obstructive pulmonary disease, *AMI* Acute myocardial infarction, *HF* Heart failure, *PHAC* Public Health Agency of Canada, *CKF* Chronic kidney failure, *CHF* congenital heart failure

Four of six included studies performed a static cross-sectional extraction of real-world data and traditional data to build their population health surveillance platform [30–33]. These studies used EHRs, claims databases, health information systems, registries, Census, social media and government and health websites and administrative hospital datasets. The *NCDCMS*—a public health data exchange platform to track NCDs in China—was updated using real-time (1-day refresh frequency) data extracted from an EHR and electronic clinical history database [35]. *PopHR* performed near real-time updates that varied in refresh frequency (2 weeks to 1 year) depending on the type of real-world data (insurance provider, retail transactions) or traditional data (Census) source [34].

### Digital aggregation of real-world and traditional data

Methods for digital aggregation of real-world data and traditional data were heterogenous across all studies. Static extracts of real-world data were aggregated via an external, independent vendor (Fine-Grained Records and Linkage Tool) and hosted in Microsoft Access [30], using a public health information network (PHIN) data system [31], via semantic web technology in a digital platform *(Social InfoButtons)* [32] or data reconciliation of pre-aggregated data *(*Canadian *CCDSS)* [33]. Real-world data were aggregated in real-time via public health data exchange with uniform data standards *(NCDCMS)* [35]. In *PopHR,* real-world data and traditional data were aggregated in near real-time using server-client architecture to execute data processing, integration and semantics [34].

### Application of aggregated data

The primary end-users of platforms developed by the included studies were public health professionals and government. *PopHR* was implemented with test users in public health and health service agencies to (a) verify software (b) generate rapid feedback and (c) conduct usability testing [34]. *Social InfoButtons* described government as an end-user to assist in disease surveillance and increase awareness of social health trends [32]. The *CCDSS* was implemented by the Public Health Agency of Canada and produces data reports, publications and interactive web-based open data resources [33]. The *NCDCMS* has been implemented in five cities in Zhejiang Province to improve public health surveillance, although the exact mechanism of this was unclear [35]. In Guilbert et al [31]., static summary reports were produced via their EHR-based HIE to describe demographic, clinic and community disease characteristics. One study transferred their deidentified and deduplicated surveillance dataset for CHD to the Centers for Disease Control for review [30].

Five of six included studies applied population health analytics to their aggregated real-world data and traditional data [31–35]. Geospatial analytics were implemented to geocode populations by disease characteristics [31, 32, 34, 35] and conduct spatial regression to geographically contextualise disease characteristics [31]. Descriptive analytics were applied across four platforms to describe disease prevalence according to multi-levelled stratification conditions (via bar charts, data tables and scatter plots) [31–34]. Temporal analytics were applied via time series to indicate population or geographical changes in prevalence over time [34], or to compare treatment efficacy in populations over time [32]. Inferential analytics were performed in the HIE by Guilbert et al [31]. to consider multivariate queries and mine relevant data characteristics. One study did not report use of analytics [30]. No studies incorporated predictive or prescriptive analytics.

### Digital health transformation towards precision public health

**Fig. 4:**
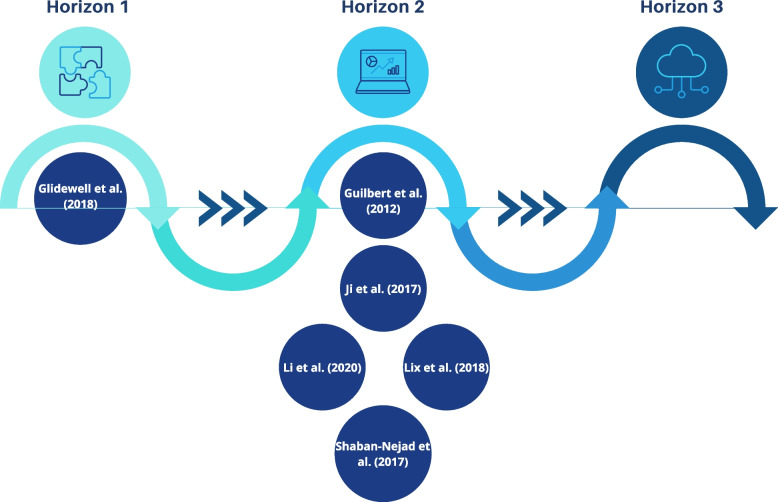
Results mapping—Three horizons towards precision public health for noncommunicable diseases

Three horizons (see Fig. 4) for digital health transformation towards precision public health for NCDs have been proposed as a strategic roadmap to guide digital public health investment, decision-making and policy [13]. One study aligned with Horizon 1 ‘Digital public health workflows’; Glidewell et al [30]. linked real-world data and traditional data and hosted the aggregate data on Microsoft Access to provide the digital foundation for improved health surveillance of people with CHD.

Five studies aligned with Horizon 2 ‘Population health data and analytics’. Shaban-Nejad et al [34]. integrated geospatial, descriptive, temporal and comparative analytics to chronic disease surveillance and tested *PopHR* in a real-world public health and health service agency setting. The social analytic tool *Social InfoButtons* provided the government with enhanced disease surveillance capability through geocoding and descriptive analytics of social and disease topic discussion online [32].

No studies aligned with Horizon 3 ‘Precision public health’.

## Discussion

### Main findings

Our systematic scoping review identified six global examples of how real-world data and traditional data is being aggregated to support precision public health for NCDs. We discovered three key findings relevant to our research question:

1. There are strong examples of aggregating real-world data and traditional data to create new knowledge about NCD prevalence and their determinants in large geographical areas in high-income countries.
2. Included studies built innovative population health methods, tools and analytics for precision surveillance of NCDs;
3. There was limited evidence of translating NCD surveillance new precision public health models of care, improved decision-making or routine public health workflows. Implementation and evaluation of population health analytics must be a priority for future work.

### Comparison to current state

Traditional decision models in public health rely upon data collected specifically for decision-making, intervention and policy for NCDs. Implementation of health information technology platforms such as EHRs is the first step to revolutionising public health by creating clinical infrastructure capacity for near real-time population health surveillance. An advanced global example is the Electronic Medical Record Support for Public Health (ESPNet) surveillance platform [17] that is the digital infrastructure for *RiskScape* – a data aggregation and visualisation platform that updates monthly to heat map disease prevalence and perform descriptive and time series statistics for various communicable (e.g. chlamydia, influenza) and noncommunicable (e.g., type 1 and type 2 diabetes, obesity, hypertension, asthma) diseases across approximately 20% of the Massachusetts (USA) population [12]. The real-world application of this platform in the Massachusetts Department of Public Health has identified NCD hot spots and risk factor targeting to inform prevention program design [12].

EHRs formed the foundation of population health surveillance systems in three of our included studies [30, 31, 35]. Variable time interval data extracts were aggregated with other real-world data (e.g. insurance providers, retail transactions) and traditional data (e.g. Census, disease registry). Recent systematic reviews have focused on the challenges (data quality, unstructured data) [19] and barriers (missing data, limited interoperability standards) [20], and solutions (natural language processing, technical solutions for data retrieval, clinical and public health collaboration) [19] and facilitators (data quality and management, preventive care) [20] to meaningful use of EHRs for surveillance. Barriers such as missing data, lack of interoperability and bias towards populations receiving care [17] may be overcome by leveraging additional real-world data to enrich the host surveillance platform.

Leveraging EHR data is an important transformative step to progressing precision public health for NCDs. EHR-based surveillance will require augmentation with other types of real-world data and traditional data from other sectors to treat the inherent challenges of use at a population scale. Aggregation of real-world data (e.g. insurance, retail transactions) and traditional data (e.g. Census) was performed by *PopHR* [34] and Guilbert et al [31]. to augment surveillance of asthma and type 2 diabetes in both examples. A “bridging” of electronic health data between the acute sector and other sectors (e.g. education, infrastructure, agriculture, transport, retail) is required to enable precision public health as NCD determinants are multifactorial, systemic and largely socially-driven [36].

Social, environmental and behavioural health determinants explain ~ 70% of health variance [22]; yet, NCD surveillance has prioritised mapping acute care and biomedical indicators. A conceptual framework for integrating the social and behavioural determinants of health into population health analytics via (a) health systems (b) non-medical organisations and (c) citizen science has been proposed in the USA to deliver comprehensive surveillance [37]. This capability will unlock precision public health and create new ethical challenges; knowledge arising from contemporaneous community data has the potential to widen health inequities if misused by certain industries. Stakeholders will retain core responsibility for ensuring ethical use of data for the public health good [38].

### Towards precision public health for noncommunicable disease

The primary advantage of a precision approach to public health is to shift the dial towards a more efficient predict-prevent system, as opposed to the currently dominant but inefficient break-fix system [7]. The foundation of advancing precision public health for NCDs is to utilise this aggregated data to deliver the right health intervention – ideally preventive or early intervention – to the right population at the right time and monitor the outcomes in a virtuous learning cycle. One pragmatic conceptual example related to obesity prevention is: community public health units access a digital dashboard containing (1) retail transactional data for fast food that is geotagged with hotspots in real-time (2) weight trajectory risk data in the early years leveraged from a statewide EMR (3) census data that augments geotagging with understanding of social position e.g. education, household income and the built environment. Then by harnessing a richly-curated, digitally-connected community, public health professionals can ‘push’ digital messages and content via social media and mHealth applications to promote healthy, family-friendly alternatives that are low cost and readily available to individuals in high-risk areas.

The true gap between current public health practice and precision public health is the progression from precision surveillance (i.e., to monitor health status) to precision action (i.e., to prevent disease). Surveillance platforms in our included studies were primarily implemented or intended for use in public health organisations, government agencies and health services to improve monitoring and reporting of chronic disease and population health and inform public health and health system planning. Evidence of their real-world application to improve public health decision-making was unclear. True precision public health for NCDs will require digital disruption of the public health sector and workflows must be optimised for data-driven, responsive and accurate decisions.

One possible reason for the gap between surveillance and action is immaturity of (a) data and (b) analytics. Most platforms identified in this review integrated analytics that favoured precision surveillance e.g., descriptive, comparative, geospatial (Horizon 2). A precision public health system is likely to be responsive to risk based on predictive (e.g., machine learning) and prescriptive (i.e., decision-support) analytics (Horizon 3) [7, 9]. One example is applying a dynamic simulation model to an NCD of interest (e.g., CVD or obesity) to quantitatively model the reach, consequences and effectiveness of various population health interventions in demographically high-risk areas. Leveraging real-world data that is updated with contemporaneity enables counting of improvement in public health that drives investment.

The digital transformation of the public health sector to prevent and manage communicable diseases is further advanced than for NCDs and was accelerated by the COVID-19 pandemic [17, 39–41]. There are lessons and opportunities from this transformation for precision public health of NCDs. Population surveillance for infectious or environmentally-driven diseases (e.g. sexually transmitted, influenza A, tuberculosis and COVID-19) is more advanced due to the requirement for fast data – transmission happens in real-time and immediate population risk is more acute, thus requiring dynamic data sources that facilitate continuous and granular population tracking. Communicable disease management requires concurrent break-fix (current state) and predict-prevent (future state) models of public healthcare. Surveillance must enable precision responsivity to outbreaks to soften their reach and impact in real-time while also enabling predictive modelling to plan resourcing, investment and policy action. The prevalence of high-burden chronic diseases (e.g., CVD, obesity, cancer) increases slowly due to compounding risk factors generated over the lifecourse. A break-fix response is necessary but problematic – once established, lifestyle-related NCDs (e.g. obesity) are difficult to reverse and attempts at reversal are often unsuccessful in the long-term [29].

### Limitations

The primary limitation of included studies was limited evaluation of population health surveillance solutions for usability and effect on public health decisions, policy and interventions. All studies were conducted in high-income countries; yet 77% of all deaths from NCDs occur in low and middle-income countries [1] where infrastructure is less digitally mature to enable precision public health. The refresh frequency of data in the surveillance platforms of included studies was mostly static and performed at cross-sectional timepoints. This generated a snapshot understanding of disease/s of interest. Lastly, some studies [30, 31, 35] concentrated on the digital architecture of solutions and missed explanation or visualisation of user experience and navigation.

The primary limitation of this review is its exclusion of population health surveillance platforms that only used EHR data for precision public health of NCDs. This was decided due to a significant body of existing review literature and to fill the clear research gap of understanding non-EHR sources of real-world data that contribute to precision public health of NCDs.

## Conclusions

Our systematic scoping review found the global focus for using real-world data and traditional data to adopt precision approaches to addressing the burden of NCDs was:

- Building digital public health foundations through integrating real-world data and traditional data into surveillance platforms (Horizon 1)
- Creating basic population health analytics as a foundation for improving policy and practice decisions (Horizon 2).

The greatest public health challenge will be uniting towards the shared vision of Horizon 3 – precision public health models of care – where data and digital technology is continuously used to improve preventive decisions and care for future consumers, populations and the public. A digital and data-driven systems approach that collaboratively includes all sectors including health, education, agriculture, transport, infrastructure, sport and recreation, finance, planning as examples is crucial to achieving this vision.

We propose the following recommendations for future research and practice in public health and health system organisations to optimise the use of real-world data and traditional data for precision public health of NCDs:

1. Integrate real-world data and traditional data to generate comprehensive surveillance reach – a ‘single source of truth’—across the social, environmental, behavioural, biomedical and commercial determinants of health.
2. Evaluate the feasibility of using population health surveillance platforms in public health settings, focusing on usability, workflow integration and impact on decision-making in the first instance.
3. Engage with consumers, communities and priority populations to explore the data-driven ethical challenges of precision public health for NCDs and their solutions.
4. Advance beyond descriptive analytics to incorporate predictive and prescriptive analytics to enable a true precision public health system.

## Supplementary Information

**Additional file 1. **Preferred Reporting Items for Systematic reviews and Meta-Analyses extension for Scoping Reviews (PRISMA-ScR) Checklist.

**Additional file 2: Supplementary Table 1.** Complete search strategy for the present scoping review according to the Population, Concept, Context framework for scoping reviews [23, 25].

## Data Availability

Not applicable.

## Abbreviations

NCD: Noncommunicable diseases
EHR: Electronic health record
PHR: Personal health record
EMR: Electronic medical record
PRISMA-ScR: Preferred Reporting Items for Systematic Reviews and Meta-Analyses – extension for scoping reviews
PCC: Population, Concept, Context
CHD: Congenital heart disease
