# Supplementary material for: Real-world data for precision public health of noncommunicable diseases: a scoping review
Source: BMC Public Health. 2022 Nov 24;22:2166. doi: 10.1186/s12889-022-14452-7 (PMC9694563; doi:10.1186/s12889-022-14452-7)
Supplement: Supplementary file 2 — Additional file 2: Supplementary Table 1. Complete search strategy for the present scoping review according to the Population, Concept, Context framework for scoping reviews [23, 25]. [file 12889_2022_14452_MOESM2_ESM.docx]

# SUPPLEMENTAL FILE 2

Supplementary Table 1: Complete search strategy for the present scoping review according to the Population, Concept, Context framework for scoping reviews^23, 25^

| Search category | Domain | EMBASE | SCOPUS | IEEE Xplore | ACM Digital Library | Google Scholar |
| --- | --- | --- | --- | --- | --- | --- |
| Population | Population and public health | 'population health'/exp OR ‘population health’:ti,ab OR 'public health'/exp OR 'public health surveillance'/exp OR 'medical informatics'/exp OR "clinical frameworks":ti,ab OR 'learning health system'/exp OR 'population surveillance'/exp OR "surveillance":ti,ab OR "platform*":ti OR ‘learning health system’:ti,ab OR 'medical information system'/exp |  | “population health” OR “public health” OR “public health surveillance” OR “medical informatics” OR "clinical frameworks" OR “learning health system” OR “population surveillance” OR "surveillance" OR "platform*" OR learning health system | “population health” OR “public health” OR “public health surveillance” OR “medical informatics” OR "clinical frameworks" OR “learning health system” OR “population surveillance” OR "surveillance" OR "platform*" OR learning health system | (“population health” OR “public health” OR “medical informatics” OR "surveillance" OR "platform*") |
| Concept | Digital aggregation | "data sharing":ti,ab OR "aggregat*”:ti,ab OR "Data network" OR linkage*:ti,ab OR "data model*":ti,ab OR 'medical information system'/exp |  | "data sharing" OR "aggregat*” OR "Data network" OR linkage* OR "data model*" OR “medical information system” | "data sharing" OR "aggregat*” OR "Data network" OR linkage* OR "data model*" OR “medical information system” | ("data sharing" OR "aggregat*” OR "data model*" OR "linkage*) |
| Context | Real-world and designed data | 'mobile application'/exp OR 'social media'/exp OR "mobile health":ti,ab OR "mobile technolog*":ti,ab OR "mhealth":ti,ab OR "m-health":ti,ab OR "billing data":ti,ab OR "claims data":ti,ab OR "data aggregation*":ti,ab OR "health data":ti,ab OR survey*:ti,ab OR "big data":ti,ab OR "digital health":ti,ab OR 'internet of things'/exp OR "Internet of Things":ti,  AND  'electronic medical record system'/exp OR 'electronic health record'/exp OR "electronic health record*":ti,ab OR "EHR":ti,ab OR "electronic medical record*":ti,ab OR "EMR":ti,ab OR "medical records":ti,ab | “mobile application” OR “social media” OR "mobile health" OR "mobile technolog*" OR "mhealth" OR "m-health" OR "billing data" OR "claims data" OR "data aggregation*" OR "health data" OR survey* OR "big data" OR "digital health" OR "Internet of Things"  AND  “electronic medical record system” OR “electronic health record” OR "electronic health record*" OR "EHR" OR "electronic medical record*" OR "EMR" OR "medical records" | “mobile application” OR “social media” OR "mobile health" OR "mobile technolog*" OR "mhealth" OR "m-health" OR "billing data" OR "claims data" OR "data aggregation*" OR "health data" OR survey* OR "big data" OR "digital health" OR "Internet of Things"  AND  “electronic medical record system” OR “electronic health record” OR "electronic health record*" OR "EHR" OR "electronic medical record*" OR "EMR" OR "medical records" | “mobile application” OR “social media” OR "mobile health" OR "mobile technolog*" OR "mhealth" OR "m-health" OR "billing data" OR "claims data" OR "data aggregation*" OR "health data" OR survey* OR "big data" OR "digital health" OR "Internet of Things"  AND  “electronic medical record system” OR “electronic health record” OR "electronic health record*" OR "EHR" OR "electronic medical record*" OR "EMR" OR "medical records" | (“population health” OR “public health” OR “medical informatics” OR "surveillance" OR "platform*") |
